# Functionally coherent transcriptional responses of *Jatropha curcas* and *Pseudomonas fragi* for rhizosphere mediated degradation of pyrene

**DOI:** 10.1038/s41598-024-51581-y

**Published:** 2024-01-10

**Authors:** L. Paikhomba Singha, K. Malabika Singha, Piyush Pandey

**Affiliations:** 1 Department of Microbiology, Assam University, Silchar, Assam 788011 India; 2 Present Address: Department of Microbiology, Central University of Rajasthan, Ajmer, Rajasthan 305817 India

**Keywords:** Microbiology, Environmental microbiology

## Abstract

Pyrene is an extremely hazardous, carcinogenic polycyclic aromatic hydrocarbon (PAH). The plant–microbe interaction between *Pseudomonas fragi* DBC and *Jatropha curcas* was employed for biodegradation of pyrene and their transcriptional responses were compared. The genome of *P. fragi* DBC had genes for PAH degrading enzymes i.e. dioxygenases and dehydrogenases, along with root colonization (*trpD, trpG, trpE* and *trpF*), chemotaxis (*flhF* and *flgD*), stress adaptation (*gshA, nuoHBEKNMG*), and detoxification (*algU* and *yfc*). The transcriptional expression of *catA* and *yfc* that respectively code for catabolic enzyme (catechol-1, 2-dioxygnase) and glutathione-s-transferase for detoxification functions were quantitatively measured by qPCR. The *catA* was expressed in presence of artificial root exudate with or without pyrene, and glucose confirming the non-selective approach of bacteria, as desired. Pyrene induced 100-fold increase of *yfc* expression than *catA,* while there was no expression of *yfc* in absence of pyrene. The transcriptome of plant roots, in presence of pyrene, with or without *P. fragi* DBC inoculation was analysed. The *P. fragi* DBC could upregulate the genes for plant growth, induced the systemic acquired resistance and also ameliorated the stress response in *Jatropha* roots.

## Introduction

Plant rhizosphere mediated bioremediation has widely been accepted as an efficient strategy for the degradation of hydrocarbons, by the process of rhizoremediation^1,2^. For successful implementation of this, the key variables are—a rhizobacteria with PAH degradation capacity for bioaugmentation, and the host plant that should endure the stressful condition imposed by PAH polluted soil^3^. In the rhizosphere, the plants release root exudates, phenolic compounds and other nutrients which is used by the rhizobacteria as sustenance, while plants obtain growth stimulating metabolites from bacteria, in return^4^. Nonetheless, the effect of root exudate on the expression of PAH catabolic genes, which could be a critical determinant in rhizoremediation, has not been assessed previously.

The United States Environmental Protection Agency (USEPA) has listed pyrene as one of the most abundant priority pollutants, among other 16 PAHs^5^. Pyrene is highly stable four ring compound and has been opted as a model compound to study the biodegradation of carcinogenic PAHs^6^. Pyrene is also known to negatively affect the growth of plants in contaminated soils. The growth of *Picea abies* seedlings and many plant species has been retarded significantly by the presence of pyrene in soil^7–9^. However, such negative affect of pyrene on *Jatropha curcas*, could be overcome by the inoculation of *Pseudomonas aeruginosa* PDB1, which in fact, promoted the growth of *J. curcas* in presence of pyrene (80 mg kg^−1^)^10^. Nevertheless, in previous studies, the addition of pyrene plants, but significantly affected the growth attributes of Fire Phoenix plant^11^. Also, PAHs treatment induced a variety of plant stress responses such as disorder in photosynthesis, trichome and leaf deformations, accumulation of H_2_O_2_, oxidative stress, upregulation of antioxidant enzyme activities, carbon allocation, protein synthesis, signal transduction and cell death^12–17^.

In rhizoremediation, it’s crucial to understand the plant molecular response during rhizosphere mediated bioremediation of PAHs, that occurs in association with bacterial partner. Therefore, in this paper, we have described the genetic environments of xenobiotic degrading genes, plant growth promoting attributes and plant root colonizing genes of the potential PAH-degrading rhizobacteria *Pseudomonas fragi* DBC. The gene *catA* code for catechol 1, 2 dioxygenase that has a role in degradation of polyaromatic hydrocarbons particularly in conversion of catechol to cis, cis-muconic acid and the *yfc* code for glutathione-S-transferase that has function in detoxification of toxic intermediates formed during degradation of polyaromatic hydrocarbons^18,19^. The quantitative expression of catabolic genes- *catA* and *yfc* of DBC has been detected in presence of different carbon sources, including root exudates. Also, the differential gene expression of *J. curcas* with or without DBC inoculation in pyrene treated conditions has been elucidated. Finally, a correlation study has been carried out between the plant physiology and soil enzyme characteristics.

## Results and discussion

### *Pseudomonas fragi* DBC genome

**Figure 1:**
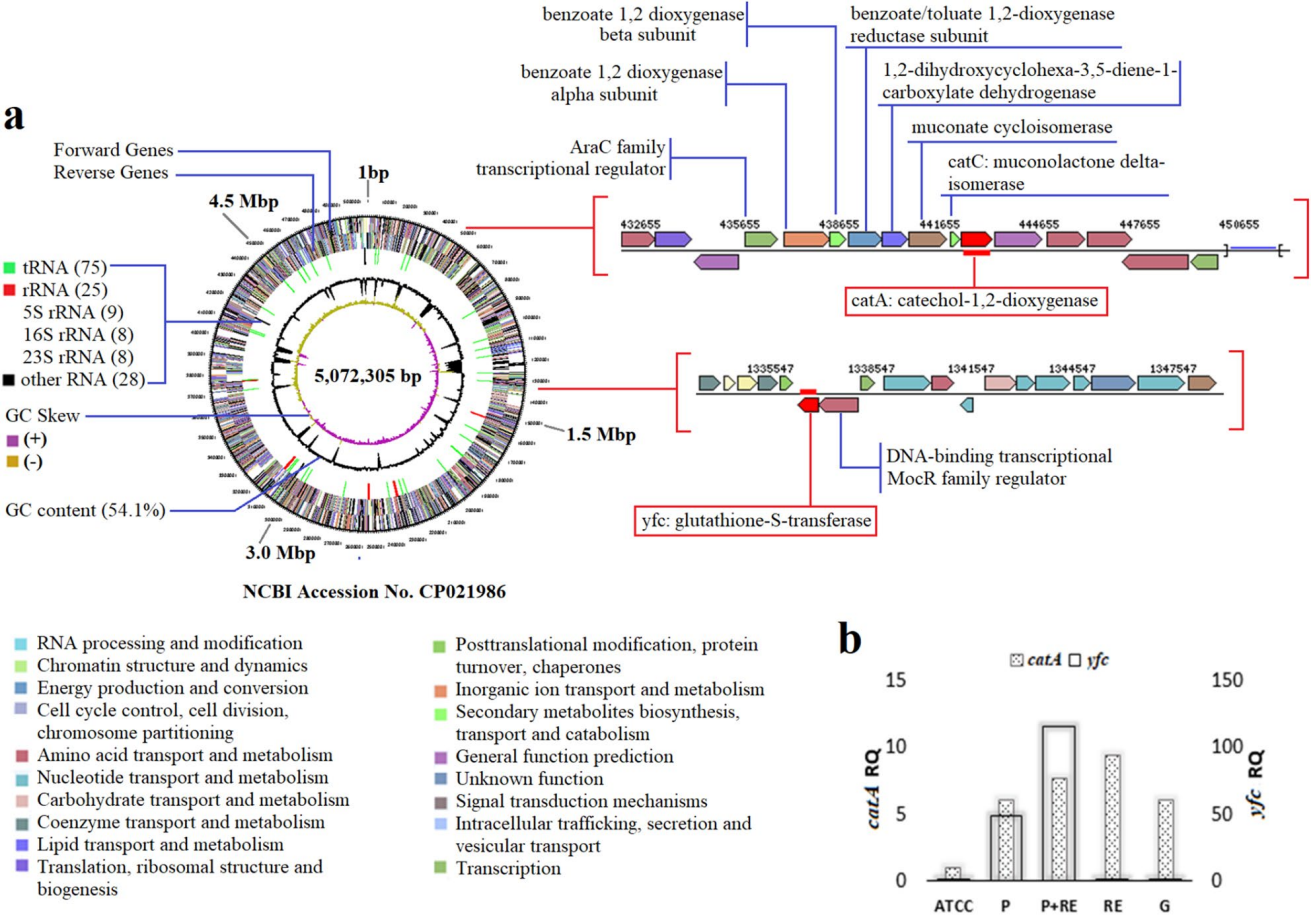
Circular draft genome map of *P. fragi* DBC. The arrangement of *catA* and *yfc* in the genome of DBC (**a**). The quantitative RT-PCR determination for *catA* and *yfc* gene expression in different carbon sources (**b**). ATCC- *Pseudomonas aeruginosa* ATCC (control for relative quantification), P-Pyrene amended BHB, P + RE (Pyrene in combination with artificial root exudates amended BHB), RE (Artificial root exudates amended BHB), and G (Glucose amended with BHB).

The *P. fragi* DBC genome annotation gave 3993 protein-coding genes, 72 tRNA genes, and 25 rRNA genes (Fig. 1a), while 88 genes were annotated to be involved in xenobiotic degradation-aminobenzoate degradation (4-phytase/acid phosphatase, alkaline phosphatase D etc.), benzoate degradation (2-oxopent-4-enoate/cis-2-oxohex-4-enoate hydratase, 3-carboxy-cis,cis-muconate cycloisomerase etc.), chloroakane and chloroalkene degradation (4-hydroxy-2-oxovalerate/4-hydroxy-2-oxohexanoate aldolase, 4-hydroxy-4-methyl-2-oxoglutarate aldolase etc.), drug metabolism—cytochrome P450 (S-(hydroxymethyl)glutathione dehydrogenase/alcohol dehydrogenase, glutathione-S-transferase) etc. (Supplementary Fig. S1). The genes for plant growth-promoting attributes and root colonization are shown in (Supplementary Fig. S2).

### Effect of root exudates on the expression of *catA* and *yfc* genes of *Pseudomonas fragi* DBC

Transcriptional expression of *yfc* and *catA* in response to different carbon sources was determined by inoculating the organisms in Bushnell Haas broth amended with either Glucose (G) or Pyrene (P) or artificial Root Exudates (RE) or artificial RE + P. The arrangement of *catA* and *yfc* in the genome of DBC and expression pattern is given (Fig. 1a). The quantitative expression of gene *catA*, which codes for catechol-1,2-dioxygenase and *yfc*, which codes for glutathione-s-transferase (GST) were determined by qPCR. The catechol-1,2-dioxygenase incorporate the molecular oxygen in to the aromatic compound through cleavage at 1st and 2nd carbon of catechol into cis,cis-muconic acid which degrades further and products finally goes in to TCA cycle. The *catA* expression was up-regulated with all carbon supplements including RE, which indicates that DBC could degrade pyrene despite the presence of other carbon sources in rhizosphere. However, *yfc* was expressed only in the treatments that had pyrene. Also, the expression of *yfc* was observed 100-fold higher than *catA* in minimal medium amended with pyrene and root exudates or pyrene amended medium only (Fig. 1b). The expression of *catA* in DBC for all treatments including RE and/or pyrene was possibly due to the absence of transcriptional regulatory gene in the genome of *Pseudomonas fragi* DBC, as reported for *Pseudomonas stutzeri* ZWLR2-1, which degrades 2-chloro, nitrobenzene constitutively, and had no NagR-like transcriptional regulator associated with dioxygenase genes^20,21^. The involvement of bacterial GST during the degradation and detoxification of various xenobiotic compounds has been reported^3,22^. Currently, *yfc*G is one of the two glutathione-S-transferase genes which has been considered as a new class of GST enzyme family (Nu)^19^, that do not act on reduced glutathione, rather more reactive to oxidized glutathione, and possess a unique disulfide bond reductase activity^23^. In the genome of DBC, Nu class of GST was annotated, as first for the genus *Pseudomonas* during this study, which had been reported previously for *E. coli*^24^.

### Transcriptome analysis of the plant roots in presence of bacteria treated with pyrene

We set up a pot experiment for rhizodegradation of pyrene by DBC, using Jatropha as host, and performed whole transcriptome analysis of DBC—treated and untreated plant. A total of 7017 and 7669 CDS of DBC treated and untreated samples respectively were found to be categorized into 23 KEGG pathways under five main categories: Metabolism, Cellular process, Genetic information, Environmental information, and Organismal systems (Supplementary Table S1).

**Figure 2:**
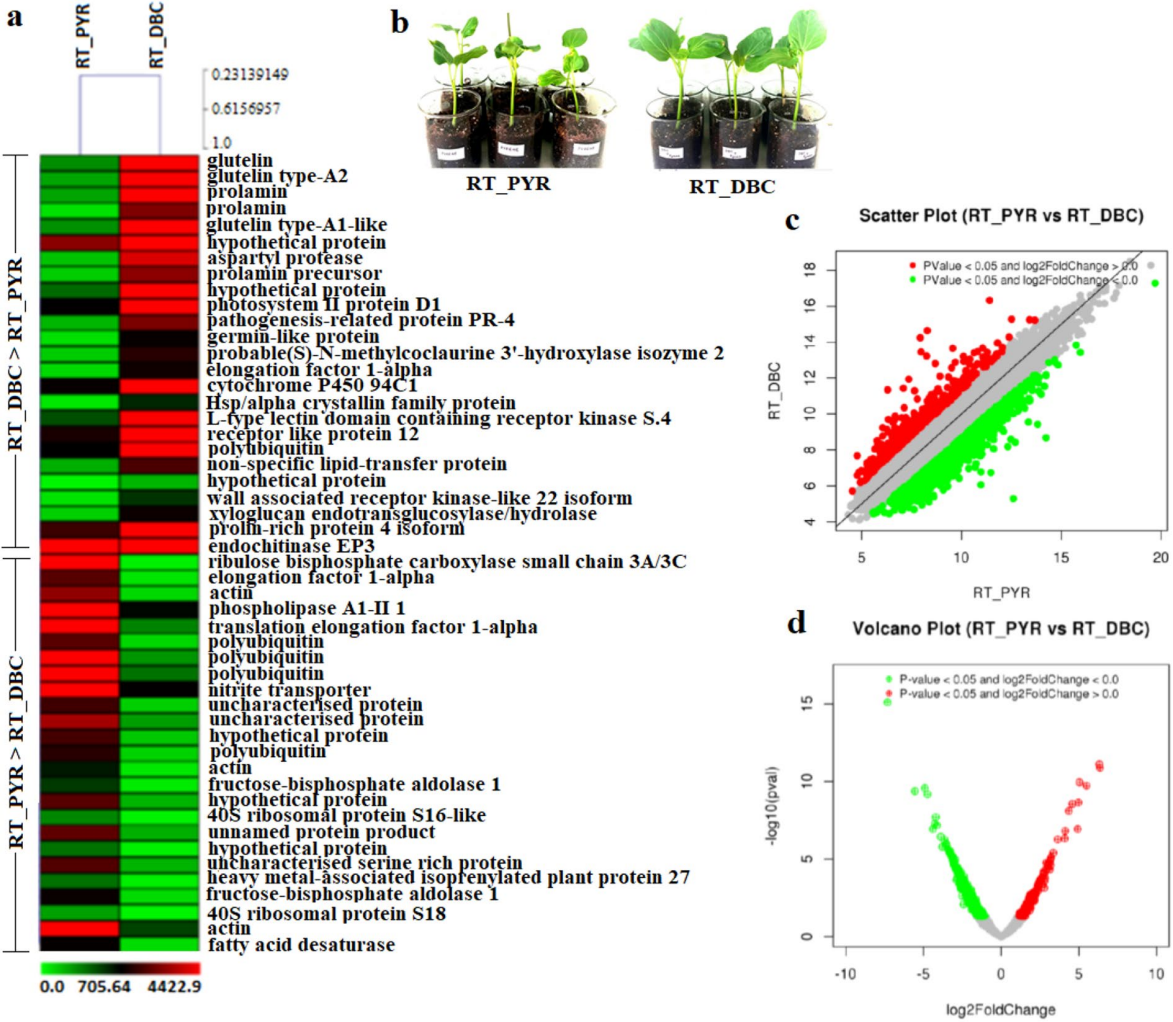
Transcriptome profile of *Jatropha curcas* in two different conditions—DBC treated (RT_DBC) and DBC untreated (RT_PYR). (**a**) Top 50 highly differentially expressed genes. (**b**) The *J. curcas* under 2 different conditions- DBC treated and DBC untreated. (**c**) Scatter Plot. The Scatter Plot is useful for representing the expression of genes in two distinct conditions of each sample combination i.e., DBC untreated and DBC treated. It helps to identify genes that are differentially expressed in one sample to another and also allows comparing two values associated with genes. In the scatter plot, each dot represents a gene. The vertical position of each represents its expression level in the Control samples while the horizontal position represents its expression level in the Treated samples. Thus, genes that fall above the diagonal are over-expressed and genes that fall below the diagonal are under-expressed as compared to their median expression level in the experimental grouping of the experiment. (**d**) Volcano plot. The 'volcano plot' arranges expressed genes along dimensions of biological as well as statistical significance. The red block on the right side of zero represents the up-regulated genes whereas green block on the left side of zero represents significant down-regulated genes. While Y-axis represents the negative log of the p-value (p < 0.05) of the performed statistical test, the data points with low p-values (highly significant) appear towards the top of the plot. Grey block shows the non-differentially expressed genes.

The top 50 highly differential expressions were observed between the DBC treated and untreated plant roots (Fig. 2). The up-regulated functions in DBC treated plant roots were glutelin, glutelin type-A2, prolamin, glutelin type-A1, aspartyl protease, photosystem II protein PR-4, germin like protein, probable(S)-*N*-methylcycloclaurite3ʹ-hydroxylase isozyme 2, elongation factor 1-alpha, cytochrome P450 94C1, Hsp/alpha cystallin family protein, L-type lectin domain containing receptor kinase S, polyubiquitin, hypothetical protein, wall associated receptor kinase-like 22 isoform, xyloglucan endotransgluocosylase/hydrolase, prolin-rich protein 4 isoform. *P. fragi* DBC induce the genes for glutelin and prolamin protein in roots. Among different seed storage protein (SSP), Glutelin content is highest in rice endosperm that contains more essential amino acid^25^. Prolamin 17D expresses in seed^26^ and is down regulated in response to drought^27^. The glutelin and prolamin genes were down regulated in the plants treated with pyrene in absence of bacteria. But the plants when inoculated with *P. fragi* DBC in presence of pyrene, showed 100–1000-fold increased production of the glutelin and prolamin. This was one of the physiological mechanisms because of which, inoculation of DBC enhanced the plant growth, despite the stressful condition levied by pyrene. Germin-like proteins (GLPs) are plant glycoproteins and a crucial component of plant basal host resistance^28^. In this study, over expression of germin-like proteins (GLPs) in the plants inoculated with DBC was noticed which was 100 fold higher than the plants without bacterial inoculation in pyrene treated conditions**.** GLPs represents a diverse and a ubiquitous plant glycoprotein in various plants^29^. GLPs are popularly known as a pathogenesis-related (PR) protein^30^. Due to their SOD or OXO activity that exhibit resistant to infection and lead to generate H_2_O_2_ during the process^31^. These H_2_O_2_ induce systemic acquired resistance of non-inoculated tissues that could initiate salicylic acid (SA) and/or jasmonic acid (JA) signalling pathways, leading to pathogenesis-related (PRs) protein synthesis and plant defences, respectively^32^. Hence, overexpression of GLPs due to inoculation of DBC furnished there was induction of systemic acquired resistance in the host plant when inoculated with bacteria.

Increased expression of PR4 along with other PRs in *Arabidopsis* activated Jasmonic acid (JA) pathway^33^. This protein served as defence response to bacteria in *J. curcas*. In present study, the 100–1000 fold up-regulation of these genes were observed in *Jatropha curcas* in presence of *P. fragi* DBC. Similarly, aspartyl protease seems to play a major role in plant–microbe interaction as there was over expression of this protein in the plants inoculated with *P. fragi* DBC than the plants without bacteria. The infection of *Arabidopsis* plants with *Pseudomonas* strain (Pst/AvrRpm1) resulted accumulation of aspartyl protease in the apoplast^34^. In healthy plants, both SAR and SA induced resistance were repressed due to overexpression of aspartyl protease without affecting the bacterial growth^35^. This enzyme also plays in maintaining the homeostatic mechanism through restriction of SAR signalling and thus modulate the resource allocation in the dealing between plant growth and defence^36^.

DBC also aided *J. curcas* for detoxification and metabolism of pyrene via induction of cytochrome P450 94C1. Yan et al.^37^ reported that the relative expression of five P450 genes—CYP81E1, CYP94C1, CYP94B3, CYP714C1, and CYP714C2 were up-regulated and while other four P450 genes—CYP97B2, CYP707A7, CYP711A1, and CYP724B1 were down-regulated during treatment of penoxsulam (herbicide). The involvement of different P450s could be a possible function in metabolizing specific herbicides^37^. Here, the cytochrome P450 94C1 was specifically induced by 100-fold increase in the plants inoculated with *P. fragi* DBC than the plants without DBC, indicating that bacterial inoculation helps plants in detoxification and metabolism of pyrene. In this study, the DBC treatment also reduced the expression of genes for phospholipase A, polyubiquitination, and heavy metal-associated isoprenylated plant proteins (HIPPs) in *J. curcas* plants treated with pyrene, which all indicated the improved plants’ ability to acclimatize the stress induced by pyrene^38^.

### Effect of pyrene on the soil enzyme and plant growth

**Figure 3:**
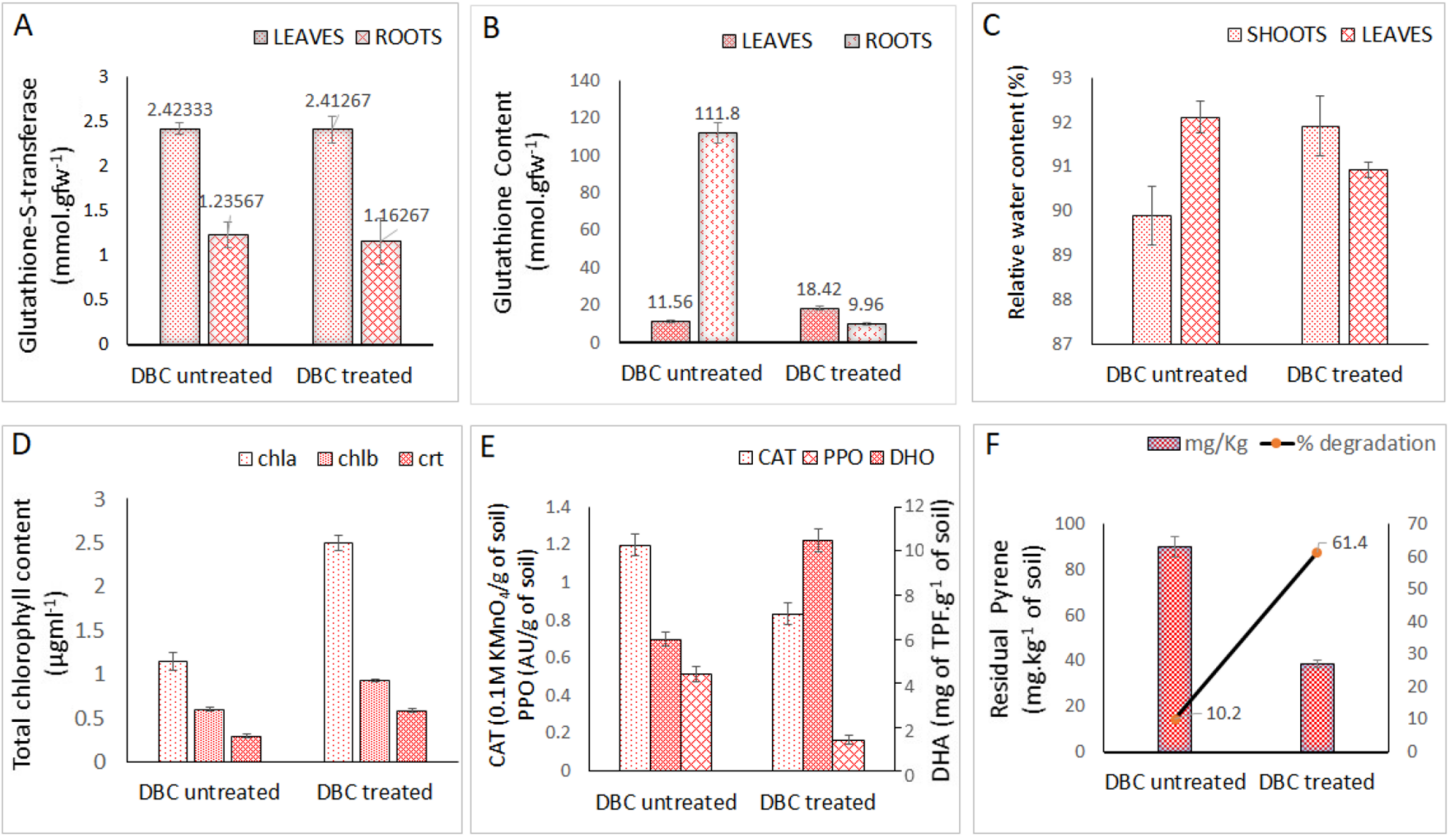
All the experiments were conducted under pyrene contaminated soils and measured after 60 days of treatment. (**A**) Glutathione-S-transferase (GST) activity of bacterial treated and untreated plants. (**B**) Glutathione (GSH) content of bacterial treated and untreated plants. (**C**) Relative water content (%), (**D**) Total chlorophyll content (mg/ml)— chlorophyll a (chla), chlorophyll b (chlb) and carotenoid (crt). (**E**) Catalase (CAT) activity, Polyphenol oxidase (PPO) activity and Dehydrogenase (DHA) activity of rhizospheric soil samples of bacterial treated and untreated plants. (**F**) Percent degradation of pyrene in rhizospheric soil of bacterial treated and untreated plants of by after 60 days of exposure.

There was no significant difference of glutathione-S-transferase activity of both the DBC treated and untreated plants under pyrene contaminated soil (Fig. 3A). DBC treated plants had shown increased RWC in shoot than leaves while in DBC untreated plants, there was lower RWC than leaves (Fig. 3C). This result suggested that the pyrene induce improper transpiration of water in leaves of the DBC untreated plants, indicating less stress of the plants in DBC treated was due to alleviation of a portion of the stress imposed upon the plant by the presence of the pyrene^39^. Also, significant increase in chlorophyll a in DBC treated than DBC untreated plants growing in pyrene contaminated soil was observed (Fig. 3D), as also previously reported for plants growing in PAH contaminated soil^40^. The glutathione content of the root in DBC untreated plants were significantly higher than DBC treated plants which is also supported with the data of transcriptome analysis (Fig. 3B). In the present study, the dehydrogenase activity was higher in the soils growing with DBC treated plant than the soils growing with DBC untreated plant. Also, the degradation of pyrene was higher in the pot with DBC treated plant, as compared with the pot of DBC untreated plant (Fig. S4F). The results suggest that PAH degrading *P. fragi* DBC can enhance dehydrogenase activity in soil and pyrene remediation efficiency (Fig. 3E). Similarly, the DHA activity in the rhizosphere was significantly enhanced by the inoculation with *Pseudomonas mendocina* compared with the other soils^41^. Xun et al.^42^ stated that the soil dehydrogenase activity is found to be negative correlation with the amount of oil pollution. Also, the correlation between the plant growth and soil enzyme in presence of pyrene is given in Supplementary Fig. S3. The strong positive correlation between dehydrogenase and plant growth parameters (total chlorophyll content and shoot RWC) suggested that DHA may be used as an indicator for the assessment of soil and plant health. The soil catalase activity was affected by the degradation activity of soil microorganisms and plant roots^14^. Xiao et al.^43^ reported that the CAT activities were increased initially then decreased gradually in all different treatment conditions. In this study, the soil catalase activity was found to be significantly high in untreated pot than treated pot condition (Fig. 3E). Here, pyrene induced the increased production of catalase in plants while inoculation of bacteria reduced it. The higher catalase activity indicates that the plants respond to the stress of PAHs as this enzyme is an antioxidant to highly ROS released during the stress conditions. Polyphenol oxidase (PPO) had been suggested for its role in hydroxylation of aromatic rings that finally lead to the mineralization of PAHs^31^. The ryegrass and bacterial combinations applied for petroleum hydrocarbon degradation in soil, had also resulted in higher PPO activities in various treatments than the control^44^, which is in accordance with our observations (Fig. 3E). Chen et al.^45^ had found that there is positive correlation between the increase activity of PPO due to higher PAH concentration in the soil^6^. Also in the present study, the residual pyrene concentration and PPO in untreated plants were higher than the DBC treated plants. The percent degradation of pyrene was 61.4% in the DBC treated pot, while it was only 10.2% in the DBC untreated pot (Fig. 3F). The soil without plant and bacteria less than 1% removal of pyrene was detected which was negligible and no significant (not presented in the Fig. 3F). The strong positive correlation between the PPO, CAT, and RGSH confirmed that the plants had to suffer the pyrene stress in the DBC untreated conditions (Supplementary Fig. S3), which was ameliorated with the treatment of PAH degrading bacteria DBC, that also facilitated removal of pyrene from the plant rhizosphere.

## Conclusion

The genome of *P. fragi* DBC possess 88 genes for degradation of various xenobiotic compounds, and also had several genes involved in plant growth promoting attributes including root colonization. The *catA*, which encode catechol-1,2-dioxygenase, was constitutively expressed despite the presence of different carbon sources including glucose and those in root exudates, making this bacterium highly suitable for rhizoremediation of pyrene, as pyrene degradation will not be affecting by presence of other substrates in soil. The expression of *yfc* was highest when pyrene (only) was given as substrate, that showed its primary role in pyrene detoxification by *P. fragi* DBC. This bacterium induced the expression of selected genes in host plant, which mainly have functions for systemic acquired resistance, xenobiotic degradation and plant growth enhancement. Therefore, *P. fragi* DBC showed a significant positive impact on pyrene removal, and in improving the plant growth upon exposure to pyrene. There was 100 to 1000-fold increase in expression of of PR4, and aspartyl protease genes in the plants treated with *P. fragi* DBC which are known to have important role in the plant symbiosis with bacteria. Overall, the experimental evidence suggests that *P. fragi* DBC—*J. curcas* combination could be used for pyrene removal from soil, as supported by the gene expression data for plant and bacterial responses that complement to facilitate the rhizosphere mediated degradation process.

## Methods

### PAH degrading Bacteria and its characteristics

*Pseudomonas fragi* DBC (NCBI Acc. No. KY593171) is a proficient PAHs degrader and could also promote the growth of host plant under PAH stress condition^46^. DBC has shown excellent degradation of pyrene and have efficient plant growth promoting attributes such as Indole acetic acid (IAA), siderophore production, phosphate solubilization, nitrogen fixation and ACC deaminase production, in presence of pyrene. This bacterium is a facultative anaerobe, grows in pH range from 6 to 8 and optimum temperature at 30 °C. The bacterium is now available in National Centre for Microbial Resource, Pune, India for public access (Accession No. MCC 3580).

### Genome analysis and functional annotation

Illumina HiSeq 2500 platform was used to obtain the draft genome sequence of the *P. fragi* DBC^10^ and the submitted to NCBI (Acc. No. CP021986). Bacterial gene prediction (PGP attributes, chemotaxis, root colonization) and functional annotation were done initially using Bacterial annotation system server 5 (BASys). The Integrated Microbial Genome (IMG) server (http://img.jgi.doe.gov) pipeline was used as a major source for genome predictions and comparison. The xenobiotic degradation genes were annotated and the pathways of selective compounds were interpreted using KEGG pathway chart.

### Expression of bacterial *catA* and *yfc* by quantitative real time PCR in presence of different carbon sources

The *yfc* and *catA* genes were predicted from the genome using IMG server and the set of primers were created by online tool GenScript. Presence of *yfc* and *catA* was confirmed by PCR assay using primers—*yfc* (pf_*yfc*F_F 5ʹ-CCTTGATCCTGCAACACTCG and pf_*yfc*F_R 5ʹ-AGCGTTCGTTCAGGTATTCG-3ʹ), *catA* (pf_*catA*_F 5ʹ-CATTATCACCGACGCCGAAG-3ʹ and pf_*catA*_R 5ʹ-CCCAGTTGATCGAGGCATTC). The artificial root exudates (RE) composed of 50 mM in stock solution—glucose, fructose and sucrose, 25 mM—succinic acid and malic acid, 12.5 mM—arginine, serine and cysteine was prepared by the method of Joner et al.^47^. The real time PCR was performed on four sets of treatments in triplicates. These are—(1) Pyrene as carbon source in Bushnell Haas Broth (BHB), (2) Pyrene in combination with artificial root exudates (RE) as carbon source in BHB, (3) RE as only carbon source in BHB, and (4) Glucose as carbon source in BHB. Relative quantification of the q-PCR was done using a *Pseudomonas aeruginosa* ATCC as a control grown in normal glucose medium. The mRNA was isolated from 21-day old culture using RNeasy Kit (Qiagen, Germany) and converted to cDNA by QuantiTect® reverse transcription kit (Qiagen, Germany). The quality of the total isolated mRNA was checked on NanoDrop (Absorbance 260/280 = 2.07). The transcriptional level of mRNA was checked using SYBRGreen PCR Master Mix (Applied Biosystems, Foster City, CA) in Step one plus real time PCR system (Applied Biosystem, USA). The method of ΔΔCt was used to quantify the relative gene expression. For the normalization of the target genes transcriptional level, the parallel expression of the 30S ribosomal gene *rpsL* was determined and calibration were done against corresponding mRNA expression by *P*. *aeruginosa* ATCC growing in glucose amended minimal medium. Relative quantification was done using a *Pseudomonas aeruginosa* ATCC grown in normal glucose medium.

### Plant material preparation, treatment, and growth condition

The plant material preparation and growth condition were followed as described previously^48^. The seeds of *Jatropha curcas* were procured from North East Developmental Finance Corporation Limited (NEDFi), India, as per the guidelines. The seeds were surface sterilized and germinated in sterilized soil rite at 30 °C in the dark. The seeds of *Jatropha curcas* were subjected to surface sterilization by immersing them in a 2% NaOCl solution for 10 min. The seeds were then washed three times using sterile distilled water and subsequently placed in sterile soil to begin the germination process. The collection of plant material was done as per IUCN Policy Statement on Research Involving Species at Risk of Extinction and the Convention on the Trade in Endangered Species of Wild Fauna and Flora, though *J. curcas* has been listed as Least Concern in The IUCN Red List of Threatened Species in 2018. Seedlings with uniform growth were selected and grown in fresh beaker containing sterilized soil rite in two different treatment conditions—DBC treated (seedlings treated with *P. fragi* DBC) and DBC untreated (seedlings with no *P. fragi* DBC treatment) under pyrene exposure. Here, *P. fragi* DBC was cultivated in nutrient broth and placed in an incubator at 30 °C while being agitated at 140 rpm for a period of 72 h. Following this, 5 mL of culture was transferred into fresh medium (45 mL) and incubated until the exponential growth phase was achieved. For *P. fragi* DBC treatment onto the root surface, the root soaking method described by Liu et al. was employed with slight modifications^49^. The bacterial cells were collected and subjected to three washes with sterile saline solution. Subsequently, the cell suspensions were adjusted to an absorbance between 0.3 and 0.4 at 600 nm using a biospectrophotometer (Eppendorf). The seedlings were dipped with bacterial suspension for 1 h at 30 °C under Laminar Air Flow. The DBC treated and DBC untreated seedlings were transferred to the beaker containing soil rite spiked with pyrene. For this, a solution of pyrene was prepared in ethyl acetate, which was then mixed with water to achieve 50 mg 100 ml^−1^ of pyrene solution. This solution was mixed with 1 kg of soil rite. After complete evaporation of ethyl acetate, the seedlings were grown for 5 days in growth chamber under sterile conditions (as described below). These two treatments were conducted in six replications (Fig. 2b).

### cDNA library construction, quantity, and quality check

The total RNA was isolated from the root tissues of DBC treated and DBC untreated plant samples using modified c-TAB and lithium chloride method^50^. The qualities and quantities of the isolated RNA samples were checked on 1% denaturing RNA agarose gel and NanoDrop, respectively. The QC passed RNA samples were subjected to prepare the RNA-Seq paired end sequencing libraries with the help of TrueSeq Stranded mRNA sample Prep kit. AMPure XP beads was used to purify ds cDNA, with the addition of A-tailing, adapter ligation and thereby enhanced through limited no. of PCR cycles. Both the Quantity and quality check (QC) of the PCR enriched libraries were checked on Agilent 4200 Tape Station.

### Cluster generation and sequence filtering

The PE illumina libraries were generated from the Qubit library concentration and Agilent Tape Station profile were loaded onto NextSeq500 for the generation of cluster and sequence filtering. Paired end sequencing of both the forward and reverse sequences of the template fragments were done on NextSeq500. The samples were bound to complimentary adapter oligos on paired-end flow cell using the kit reagents. By using Trimmomatic v0.38 and in house script, the high-quality concordant reads were obtained from sequenced raw data for the samples by removing adapters, ambiguous reads (reads with unknown nulceotides “N” larger than 5%), and low-quality sequences (reads with more than 10% quality threshold (QV) < 20 phred score). For de novo assembly, the resulting high quality (QV > 20), paired-end reads were used to assemble into transcripts with the help of Trinity de novo assembler (version 2.8.4). The resulted sequences, unigenes that have > 90% coverage at 3X read depth were taken for downstream analysis.

### Coding sequence prediction and functional annotation

The unigenes were predicted for coding sequences using TransDecoder-v5.3.0. DIAMOND (BLASTX alignment mode) was used to find the homologous sequences of the genes against non-redundant protein database from NCBI for functional annotation of genes. Majority of the blast hits were found to be against *Jatropha curcas.* For identification of the potential involvement of the predicted CDS in biological pathways, CDS from the RT_DBC and RT_PYR samples were mapped to reference canonical pathways in KEGG (Eukaryotes database)^51^.

### Differential gene expression analysis

A total of 4.89 GB and 4.54 GB high quality paired reads for DBC treated and untreated samples respectively were pooled together. A total of 89,133 non-redundant validated unigenes were considered for further downstream analysis. The predicted CDS were subjected to functional annotation using DIAMOND (BlastX mode) against the NCBI 'Nr' database. The analysis of differential gene expression was done on the CDS between DBC treated and DBC untreated samples by the application of DESeq package (a negative binomial distribution model). The calculation of Log2 fold change (FC) value on the read abundance was done by using the formula: FC = Log2 (Treated/Control). The upregulated and downregulated genes are considered according to the CDSs having log2 fold change value greater than zero and less than zero respectively. To filter statistically significant results, P-value threshold of 0.005 was used.

### Preparation of PAHs-contaminated soil and plant materials

The pots were filled with 1 kg soil rite spiked with 100 mg kg^−1^ of Pyrene as described previously^52^. The pyrene was added initially to 100 g of soil rite followed by mixing the whole amount of soil thoroughly to get homogeneous mixture of pyrene. The *J. curcas* seeds were germinated as described above and the seedlings of equal size are planted under sterilized soil rite in two different treatments—DBC untreated (Plants treated without *P. fragi* DDBC under pyrene exposure), and DBC treated (Plants treated with *P. fragi* DBC under pyrene exposure). The determination of residual amount of pyrene by GCMS has been conducted after 60 days of treatment as described in our previous report^48^. To estimate the pyrene content, the soil samples (20 g) that were collected from the pots were placed into a conical flask. They were then combined with ethyl acetate, specifically 20 g of soil mixed with 40 ml of ethyl acetate. GC–MS analysis was carried out utilizing the Clarus 680 Gas Chromatograph and the Clarus 600C MS mass spectrometer system.

### Growth chamber condition and sterility check

The seedlings were grown for 60 days within a controlled environment in a growth chamber. The conditions within the growth chamber consisted of a photon flux of 220 μmol photons m^−2^ s^−1^, with a 16-h photoperiod, maintained at temperatures of 30/26 ± 2 °C during day and night, and a relative humidity of 65 ± 5%. To ensure the prevention of any unintended contamination, rigorous precautions were taken. The experiment was executed in triplicate for each independent trial. Prior to the initiation of experiments, the growth chamber was subjected to fumigation three days in advance. The ventilation ports were sealed to prevent the ingress of insects or other external contaminants. Throughout the entire experiment, all pots were exclusively irrigated with sterile water.

To confirm the sterility of the experimental setup, a sterility check was performed using pots containing sterile soil as described in previous report^48^. This sterile soil served as a control to verify the absence of cross-contamination. At 15-day intervals, a suspension of soil (consisting of one gram of soil prepared with sterilized water) was plated on a nutrient agar medium, and colony-forming units (CFU) were monitored. No CFUs were detected in these checks, and therefore, no specific data is provided regarding CFU counts.

### Chlorophyll content

The 0.5 g of fresh plant leaf was homogenized by using mortar and pestle with 10 ml of acetone and centrifuged at 10,000 rpm (4 °C) for 15 min. 0.5 ml of supernatant were mixed with 4.5 ml of the acetone and analysed for Chlorophyll-a, Chlorophyll-b and carotenoids content in UV–Vis spectrophotometer (Shimadzu, UV-2550, Kyoto, Japan). The equation used for the quantification of Chlorophyll-a, Chlorophyll-b, and carotenoids was used as given by Sumanta et al.^53^.

### Relative water content

The plant’s physiological attributes were measured and compared by taking biomass weight (fresh and dry), relative water content (RWC) and growth length (shoots and roots) according to Singha et al.^46^. The measurement of plant’s growth such as shoot length, root length, leaflets and the total biomass were also taken in triplicate.

### Estimation of plant GST activity and GSH content

Reduced glutathione (GSH) of the plants leaves and roots were determined according to Moron et al.^54^. The Glutathione-S-transferase (GST) activity was measured according to the method of Habig et al.^55^. The estimation of GST activity was done as suggested by Singha et al.^46^.

### Soil enzyme analysis

The activity of soil dehydrogenase (DHA) was measured by classical triphenyl tetrazolium chloride method^56^. The activity of DHA was measured as μg TPFg^−1^ h^−1^. The soil polyphenol oxidase (PPO) was measured using pyrogallic acid as substrate^57^. The determination of soil catalase activity was done by KMnO_4_ titration method with H_2_O_2_ as substrate. Catalase activity was expressed in 0.1 mol L^−1^ KMnO_4_ solution titrated per gram of dry soil)^57^. All estimation of enzymatic activity was observed in triplicates.

### Statistical analysis for correlation

Data from soil and plant enzymes were performed by Analysis of Variance (ANOVA) with SPSS Statistics Software version 19 (IBM), with a P value < 0.05 significant difference. The depiction of the graphical representation and distribution of differentially expressed genes was done using the proprietary R script. Correlation analysis was conducted among the plants and soil parameters using R studio.

### Supplementary Information

Supplementary Information.

## Data Availability

Sequences were deposited in the NCBI under accession numbers KY593171, CP021986, SRR10083544, SRR10083543.
