# Supplementary material for: Functionally coherent transcriptional responses of Jatropha curcas and Pseudomonas fragi for rhizosphere mediated degradation of pyrene
Source: Sci Rep. 2024 Jan 10;14:1014. doi: 10.1038/s41598-024-51581-y (PMC10781960; doi:10.1038/s41598-024-51581-y)
Supplement: Supplementary file 1 — Supplementary Information. [file 41598_2024_51581_MOESM1_ESM.docx]

**Supplementary File**

**Transcriptional harmony between *Jatropha curcas* and *Pseudomonas fragi* for pyrene rhizodegradation**

L. Paikhomba Singha, K. Malabika Singha, Piyush Pandey^*^

Dept. of Microbiology, Assam University, Silchar, Assam, India

* For correspondence: piyushddn@gmail.com; ppmicroaus@gmail.com

Supplementary data-Fig. 1S, 2S and 3S

**Xenobiotic degradation genes in *Pseudomonas fragi* DBC.** The first version of annotation includes 3,993 protein-coding genes, 72 tRNA genes, and 25 rRNA genes (Fig 1a). In the *P. fragi* DBC, a total of 88 genes were annotated to be involved in xenobiotic degradation- aminobenzoate degradation (4-phytase/acid phosphatase, alkaline phosphatase D, amidase, enoyl-CoA hydratase, gallate dehydrogenase), atrazine degradation (Urease alpha, beta, gamma subunit genes), benzoate degradation (2- oxopent-4-enoate/cis-2-oxohex-4-enoate hydratase, 3-carboxy-cis,cis-muconate cycloisomerase, 3-oxoadipate enol-lactonase, 3-oxoadipyl-CoA thiolase, 4 hydroxy-4-methyl-2-oxoglutarate aldolase, oxalomesaconate hydratase, 4-hydroxybenzoyl-CoA thioesterase, 4-oxalomesaconate tautomerase, acetaldehyde/propanal dehydrogenase, acetyl-CoA acyltransferase, benzoate/toluate 1,2-dioxygenase alpha subunit, benzoate/toluate 1,2-dioxygenase beta subunit, benzoate/toluate 1,2-dioxygenase reductase subunit, catechol 1,2-dioxygenase, dihydroxycyclohexadiene carboxylate dehydrogenase, dehydrogenase, enoyl-CoA muconate cycloisomerase, hydratase, muconolactone glutaryl-CoA D-isomerase, p-hydroxybenzoate 3-monooxygenase, protocatechuate 3,4-dioxygenase alpha subunit, protocatechuate 3,4-dioxygenase beta subunit), chloroakane and chloroalkene degradation (4-hydroxy-2-oxovalerate/4-hydroxy-2-oxohexanoate aldolase, 4-hydroxy- 4-methyl-2-oxoglutarate aldolase, 4-hydroxybenzoyl-CoA thioesterase, 4-oxalomesaconate hydratase 4-oxalomesaconate tautomerase acetaldehyde/propanal dehydrogenase, acetyl-CoA acyltransferase, benzoate/toluate 1,2-dioxygenase alpha subunit, benzoate/toluate 1,2-dioxygenase, beta subunit benzoate/toluate 1,2- dioxygenase, reductase subunit, catechol 1,2-dioxygenase, dihydroxycyclohexadiene carboxylate dehydrogenase, dehydrogenase, muconate enoyl-CoA cycloisomerase, hydratase, muconolactone glutaryl-CoA D-isomerase, p-hydroxybenzoate 3-monooxygenase, protocatechuate 3,4-dioxygenase alpha subunit, protocatechuate 3,4-dioxygenase beta subunit), drug metabolism - cytochrome P450 (S- (hydroxymethyl)glutathione dehydrogenase/alcohol dehydrogenase, glutathione-S-transferase, monoamine (hydroxymethyl)glutathione oxidase, naphthalene dehydrogenase/alcohol degradation dehydrogenase, (S-alcohol dehydrogenase) etc. (Fig 1d). The genes for plant growth-promoting attributes and root colonization are shown in figure 1e (Fig 1e).


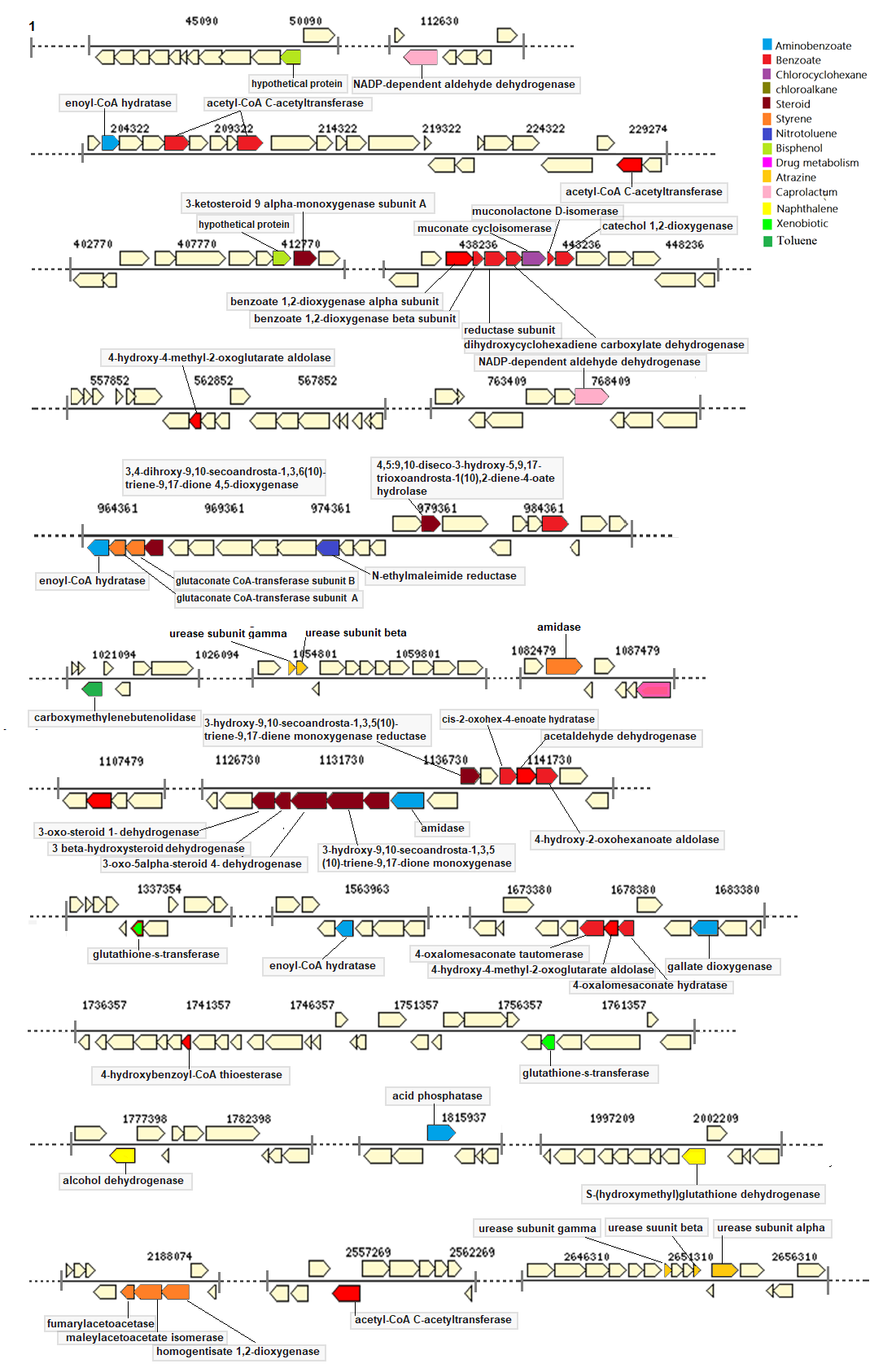


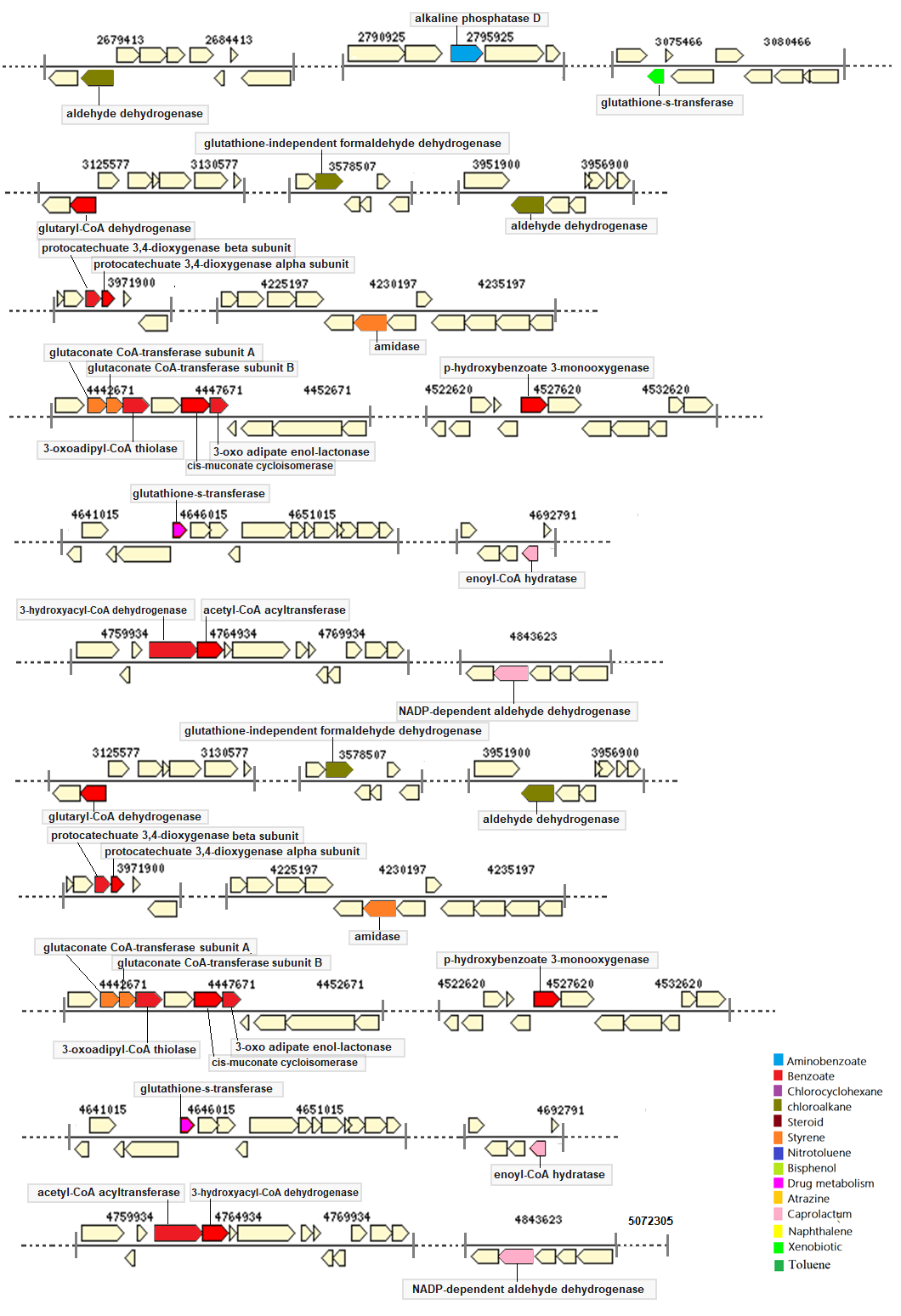


Figure S1. Xenobiotic degradation genes using Kegg pathway analysis in *P. fragi* DBC.


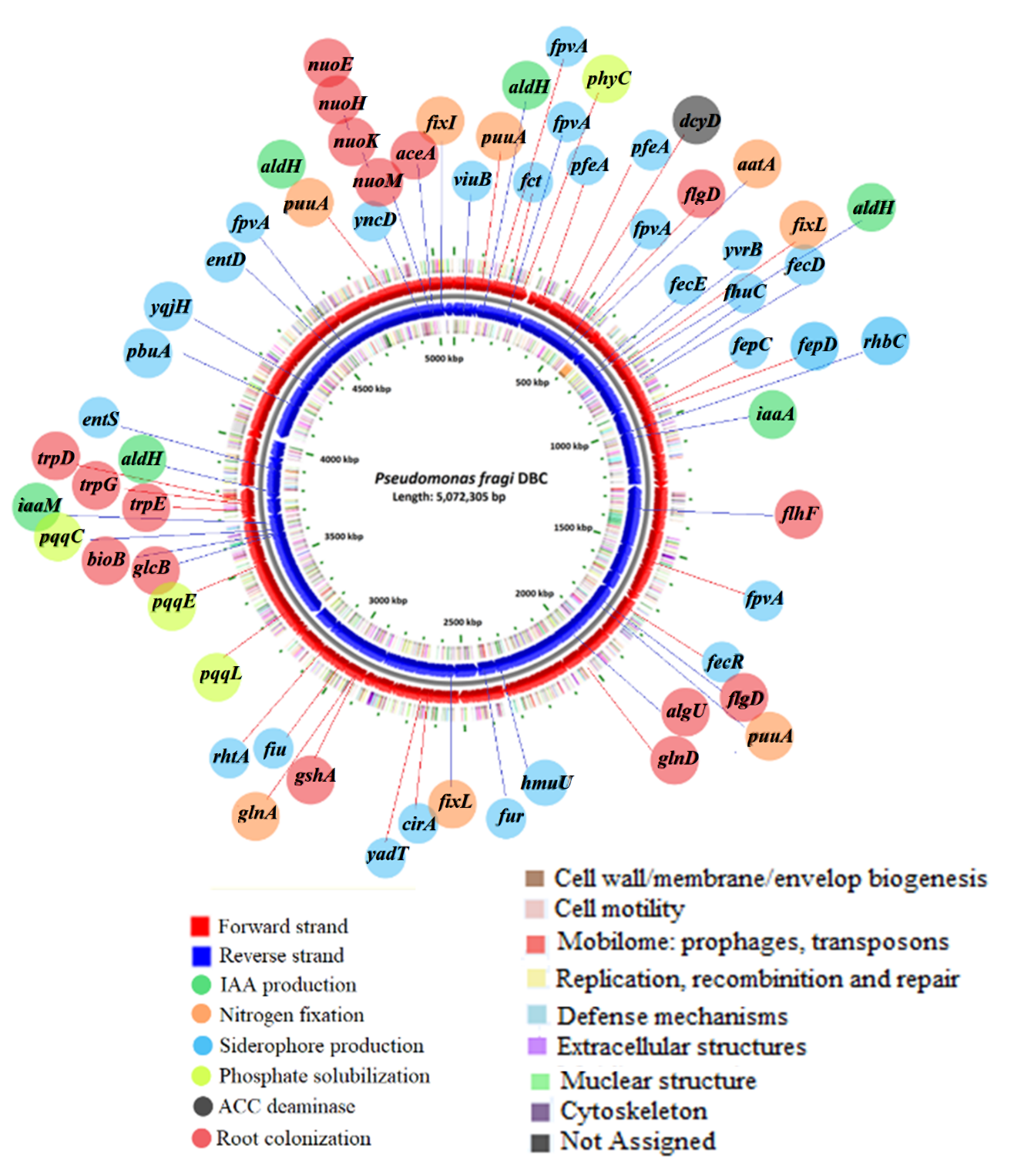


Figure S2. Basys annotation of *P. fragi* DBC plant growth promoting genes.


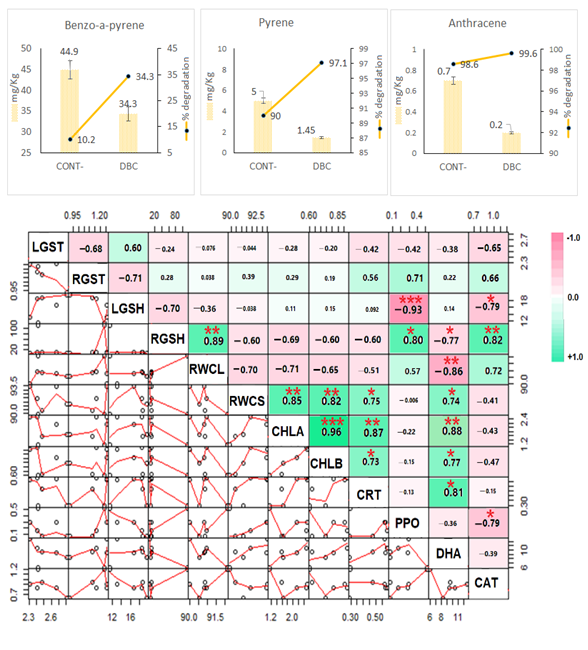


Figure S3. The correlation analysis among plant growth parameters and soil enzyme analysis

**Supplementary data-Table1**

**Transcriptome analysis of the plant roots in presence of bacteria treated with pyrene.** Whole transcriptome analysis performed on RT_PYR and RT_DBC samples. A total of 4.54 GB and 4.89 GB high quality paired reads for RT_PYR and RT_DBC samples respectively were pooled together and de novo transcriptome assembly was performed using Trinity de novo assembler followed by cd-hit-est. A total of 89,133 non redundant validated unigenes were considered for further downstream analysis. The CDS prediction was carried out using TransDecoder on the above mentioned unigenes which resulted in a total of 67,215 CDS. The predicted CDS were subjected to functional annotation using DIAMOND (BlastX mode) against the NCBI ‘Nr’ database. A total of 57,943 CDS were functionally annotated. The majority of the CDS were found to be annotated against Jatropha curcas. Out of the 67,215 CDS mentioned above, it was found that 39,615 and 45,083 CDS belong to RT_PYR and RT_DBC samples respectively. As a result of Gene ontology (GO) analysis using B2G framework, Molecular Function was found to have the highest number of CDS associated with it for all the samples. KEGG pathway analysis was carried out for all the individual set of CDS. A total of 7,669 and 7,017 CDS of RT_PYR and RT_DBC samples respectively were found to be categorized into 23 different functional KEGG pathways (Table 1). The majority of CDS were found to be associated with Translation Differential gene expression analysis was carried out after identifying the respective set of CDS for each of the samples.

**Supplementary Table S1**. Kegg pathway classification of CDS

| **Pathways** | **RT_DBC CDS count** | **RT_PYR CDS count** |
| --- | --- | --- |
|  | **Metabolism** |  |
| Carbohydrate metabolism | 737 | 642 |
| Energy metabolism | 481 | 427 |
| Lipid metabolism | 348 | 309 |
| Nucleotide metabolism | 148 | 141 |
| Amino acid metabolism | 211 | 181 |
| Cofactors and vitamins | 274 | 241 |
| Terpenoids and polyketides | 170 | 144 |
| Secondary metabolites | 198 | 172 |
| Xenobiotic biodegradation | 110 | 96 |
|  | **Genetic information Processing** |  |
| Transcription | 347 | 307 |
| Translation | 859 | 806 |
| Folding, Sorting and degradation | 587 | 556 |
| Replication and repair | 142 | 131 |
|  | **Environmental Information Processing** |  |
| Membrane transport | 39 | 36 |
| Signal transduction | 142 | 131 |
|  | **Cellular Process** |  |
| Transport and catabolism | 553 | 501 |
| Cell growth and death | 353 | 332 |
|  | **Organismal Systems** |  |
| Environmental adaptation | 384 | 349 |
